# Combined Medicine-Pediatrics Fellowships: A Guide for Fellowship Directors and Residents

**DOI:** 10.7759/cureus.15688

**Published:** 2021-06-16

**Authors:** Burton H Shen, Janaki Vakharia, Lisa S Topor, Brett Robbins, Kathryn Diamond-Falk, Stefanie Brown, Katherine Mason, Christine Barron, Debra L Simmons, Kevin M McKown, Suzanne McLaughlin

**Affiliations:** 1 Medicine and Pediatrics, Lifespan/Brown University, Providence, USA; 2 Endocrinology, Massachusetts General Hospital/Harvard University, Boston, USA; 3 Endocrinology, Hasbro Children's Hospital/Brown University, Providence, USA; 4 Internal Medicine-Pediatrics, University of Rochester Medical Center, Rochester, USA; 5 Internal Medicine-Pediatrics, Maine Medical Center/Tufts University, Portland, USA; 6 Internal Medicine, University of Miami Miller School of Medicine, Miami, USA; 7 Pediatrics, Hasbro Children's Hospital/Brown University, Providence, USA; 8 Child Protection, Hasbro Children's Hospital/Brown University, Providence, USA; 9 Endocrinology, Diabetes and Metabolism, University of Utah, Salt Lake City, USA; 10 Medicine/Endocrinology, Salt Lake City Veterans Affairs Medical Center, Salt Lake City, USA; 11 Rheumatology, University of Wisconsin, Madison, USA; 12 Medicine-Pediatrics, Lifespan/Brown University, Providence, USA

**Keywords:** combined fellowship, medicine-pediatrics, subspecialty training

## Abstract

Dual training in Internal Medicine-Pediatrics (MedPeds) was recognized by the American Board of Medical Specialties in 1967. Residents complete 24 months each in Internal Medicine and Pediatrics and are board-eligible for both at the conclusion of training. Graduates are eligible for fellowships in either or both fields. Many graduates pursue fellowship training. A small absolute number of graduates apply for dual training in adult and pediatric subspecialties, but those that do bring direct, in-depth clinical experience across the lifespan, and familiarity with care in both pediatric and adult settings. As such, they contribute unique perspectives and capabilities to their fellowship and future practice. This includes the ability to provide subspecialty care in settings with limited resources, where they are able to address needs without age restrictions, and in the transition of subspecialty care for emerging adults with childhood-onset conditions.

Due to the small number of applicants pursuing joint adult and pediatric fellowships, many fellowship directors may have limited experience with dual fellowships but may want to create opportunities for these unique trainees. This summary was developed jointly by residents, fellows, MedPeds program directors, and fellowship directors in Pediatrics and Internal Medicine subspecialties, and approved by their respective leadership councils to offer some key points on common questions, suggest additional resources, and share best practices, with a goal of facilitating this process for fellowship programs and residents alike.

## Introduction

This guidance was developed and approved by the Medicine-Pediatrics Program Directors Association (MPPDA) and the Association for Specialty Professors of the Alliance for Academic Internal Medicine, the Fellowship Directors’ Executive Committee of the Association of Pediatric Program Directors, and the National MedPeds Residents Association.

Dual training in Internal Medicine-Pediatrics (MedPeds) was recognized by the American Board of Medical Specialties in 1967. Residents complete 24 months each in Internal Medicine and Pediatrics and are board-eligible for both at the conclusion of training. The specialty has produced more than 6000 graduates over the 50 years of its existence (Agarwal A for the Research Committee MPPDA, email, May 15, 2019). Graduates are eligible for fellowships in either or both fields. Currently, approximately 1/3rd of graduates pursue fellowship training. 

A relatively small number of residents apply for dual training in both adult and pediatric fellowships. However, they bring direct, in-depth clinical experience across the lifespan, and familiarity with care in both pediatric and adult settings. As such, they contribute unique perspectives and capabilities to their fellowship and future practice. This includes the ability to provide subspecialty care in settings with limited resources, where they are able to address needs without age restrictions, and in the transition of subspecialty care for emerging adults with childhood-onset conditions.

Due to the small number of applicants pursuing joint adult and pediatric fellowships, many fellowship directors may have limited experience with dual-fellowships. Currently, publications on combined fellowships include perspective pieces on specific subspecialty experiences [1-5], descriptions of site-specific programs [6], and a single survey of graduate outcomes [7]. This summary was developed jointly by residents, fellows, MedPeds program directors, and fellowship directors in Pediatrics and Internal Medicine subspecialties, and approved by their respective leadership councils to offer some key points on common questions, suggest additional resources, and share best practices. 

## Materials and methods

Available guidance from the American Boards of Medical Specialties and National Residency Matching Programs were reviewed. We solicited the input of key stakeholders, including direct outreach to a convenience sample of trainees in or having completed combined fellowship training; program and fellowship directors at institutions offering combined training; MPPDA, the Association for Specialty Professors (ASP) of the Alliance for Academic Internal Medicine (AAIM); the Fellowship Directors’ Executive Committee of the Association of Pediatric Program Directors (FDEC APPD); and the National MedPeds Residents Association (NMPRA). Key elements were summarized by the authors and reviewed, modified, and approved by leadership groups of MPPDA, ASP, AAIM, FDEC APPD, and NMPRA. 

## Results

Logistics of combined adult/pediatric subspecialty training

The American Boards of Medical Specialties

The American Board of Pediatrics (ABP) and the American Board of Internal Medicine (ABIM) provide specific guidance on combined fellowships [8,9]. Combined fellowship training can be one year less than would be required of full training in both subspecialties, reflecting double-counting of time devoted to scholarly activity. Individuals in combined subspecialty training would be expected to meet the same clinical training requirements as those in standard internal medicine or pediatric subspecialty programs (typically, one year minimum required clinical training each for Medicine and Pediatric components; however there are exceptions, such as 18 months required for Pediatric Cardiology and Gastroenterology (GI) and 24 months for adult Cardiology and GI). 

It is important to note that the boards do not approve programs for combined training; rather, they consider training proposals for individuals. ABP and ABIM require proposals to be submitted prior to or within the first six months of the combined fellowship and to include a block diagram of training in each subspecialty, a detailed description of how the scholarly activity component will be met, and assurances that the same clinical components, including conferences, continuity clinic, and didactic teaching experiences will be met by the individual.

The Accreditation Council for Graduate Medical Education

The specialty-specific adult and pediatric guidelines should be reviewed by the adult and pediatric fellowship directors and the trainee to ensure the curriculum proposed to the ABMS meets all requirements. Here are two examples where the specialty-specific requirements may necessitate additional considerations in the overall scheduling: 

1. Research requirements may vary across the adult and pediatric programs. For example, pediatric infectious disease requirements state, “The scholarly experience must begin in the first year and continue throughout the duration of the educational program. (Core IV.D.3.d)” [10]. Adult infectious disease requirements do not specify the timing of scholarship and so fellowships may not be usually structured for a first-year fellow to start research. “The majority of fellows must demonstrate evidence of scholarship conducted during the fellowship. (Outcome IV.D.3.a)” [11]. Based on these guidelines, combined programs may need to adjust first-year schedules for fellows starting on the adult side to allow for the initiation of research projects that continue over the duration of the program. 

2. Continuity clinic requirements may vary across the adult and pediatric programs. For example, pediatric endocrinology states, “IV.C.5. Fellows must have responsibility throughout their educational program for providing longitudinal outpatient care that is supervised by one or more members of the pediatric endocrinology faculty (Core. IV.C.5) [12]. Adult endocrine requirements note a minimum of 2 half-days of ambulatory care per week, averaged over 2 years ... the continuity patient care experience should not be interrupted by more than one month, excluding a fellow’s vacation (IV.C.5)" [13]. Based on interpretation, these requirements may necessitate a combined fellow scheduling their continuity sessions in both adult and pediatric settings to occur throughout the course of the fellowship, including when rotating in the paired program. 

Institution-Specific Logistics

Willingness for collaboration between the institution’s adult and pediatric fellowship directors is necessary in recruitment and throughout the fellowship. 

At the recruiting stage, discussions prior to the match with the adult or pediatric counterpart are essential to establish the elements, sequence, and funding of fellowship training, and the appropriate Match to the pediatric or adult program. These should be agreed upon between the applicant and fellowship directors early in the process. 

After the match, a written letter of acceptance to a combined fellowship should be signed by the adult and pediatric fellowship directors and the applicant for clarity on the full commitment by both training programs. 

Creating a Schedule

A combined fellowship brings about unique scheduling challenges in order to meet both ABIM and ABP fellowship requirements. Each specialty has its own unique set of ACGME and ABMS requirements. Furthermore, each institution has unique scheduling, clinic, call, and coverage models that require consideration. There is no single “right” design for a combined fellowship schedule; the process will require ongoing collaboration with the fellowship directors and the applicant-fellow. Once a schedule is created that meets all requirements, it can likely be used as a template for any future applicants for combined fellowships. 

Some programs find the logistics most manageable with the clinical training structured sequentially. An elective each year in the alternate specialty allows for greater integration. ACGME requirements for continuity clinic, research, or other content may dictate some adjustments from a typical fellow schedule. The proposed curriculum is submitted to the ABIM and ABP within the first six months of fellowship. 

The timing of ABIM and ABP certifying exams should be considered. ABIM is scheduled typically in the second half of August and ABP in mid-October. Graduating residents need to commit to the examination dates in the spring. Fellowship Directors (FDs) should provide guidance on whether to sit on both boards in the first year and whether fellowship schedules can be arranged to accommodate the exam dates. Data indicates that pass rates are highest for first-time takers in the year of graduation, so it is generally encouraged to take exams in the first year of fellowship if schedules can ensure at least some preparation time and adequate rest beforehand. 

Managing the Match

National residency matching program (NRMP) rules regarding the fellowship match 

Applicants seeking dual-fellowship positions submit online applications via the Electronic Residency Application Service (ERAS) to both the adult and pediatric subspecialty of choice. The application and interview cycle between pediatric and adult fellowship programs overlap for most subspecialties, and dual applicants will often have the opportunity to apply and interview around the same time for both the adult and pediatric fellowship program at a given institution. Although the application and interview process for subspeciality fellowships across the adult and pediatric programs may be shared through the common ERAS platform, the match process and schedules for the Pediatric Specialties Match (PSM) and the adult Medical Specialties Matching Program (MSMP) are currently separate, and a dual applicant may only register for one match at a time during a given match cycle. Current match cycles and rank list deadlines overlap but do not align, for pediatric and adult subspecialties, with the adult match typically occurring about two weeks before the pediatric match. In order for an adult fellowship program to include an applicant in their program’s rank list, the applicant must be registered through NRMP for the MSMP, whereas for a pediatric fellowship program to include an applicant, the applicant must be registered through NRMP for the PSM. The current match structure requires a dual applicant to commit to either the adult (MSMP) or pediatric (PSM) match in order to be ranked and is a barrier to dual fellowship applicants and programs. Applicants may have individualized discussions with the pediatric and adult programs at each institution as to whether to apply solely through their pediatric or adult program with a plan of enrolling in the paired program subsequent to the match, but there is not a consistent preference within specialties nor across institutions for applying to the pediatric versus adult programs. 

As a result, applicants face significant uncertainty and potentially truncated options based on the match they opt to enter. For example, if an applicant’s first, fourth, fifth choice institution prefers that they register for the match through the PSM but their second and third choice institutions prefer registration through the MSMP, the applicant could not fulfill his/her desired rank list because he/she may only register for one match (MSMP or PSM) at a time. A common match that allows dual applicants to integrate pediatric and adult programs into one rank list, or a consensus within a given subspecialty by all institutions about which match (MSMP vs. PSM) dual applicants should use are possible long-term solutions. The former allows for more flexibility among programs of different subspecialties and at different institutions to internally determine which program, either through the adult or pediatric fellowship program, they prefer to rank the dual applicant. Considering that the Main Residency Match currently allows for the integration of multiple specialties into a rank list (i.e., an applicant can rank Pediatrics and Internal Medicine-Pediatrics programs into one rank list), this may be an achievable solution that would ameliorate one of the challenges dual fellowship applicants face.

In offering a position for a combined fellowship, the non-match adult or pediatric counterpart program is agreeing to provide a position in advance, usually for the following year, outside the match. Because the agreement occurs outside of the NRMP, there is no uniform guidance on how these agreements are set. Many applicants report that this is a verbal understanding of the plan, with emails between adult and pediatric fellowship directors and the applicant, but not a signed agreement. A written document signed by all parties would provide clarity of expectations and requirements. In some instances, this could create conflicts with a subspecialty-NRMP All In Policy. However, only a few specialties have implemented an All In Policy for fellowship match, and those that have been committed to 75% compliance. Information on the All In Policy and the specialties that have implemented it can be found at http://www.nrmp.org/all-in-policy/fellowship-matches. 

Multiple fellowship directors reported the experience of having multiple competitive applicants seeking dual fellowship positions but concerns from their counterpart program of filling more than one fellowship position with dual applicants. If more than one applicant is ranked and matched, it could be overly disruptive to their categorical staffing to have multiple fellows shifting across programs. On the other hand, if a single match position was dedicated to a dual position and no dual candidates matched (from the significantly smaller pool of dual candidates), a position would remain unfilled. A partial solution to this concern already exists: the NRMPs Reversion option. The reversion option allows a program to create distinct rank lists for combined and categorical candidates, specifying the number of positions for each type and allowing an unfilled position to “revert” to the available categorical positions and be filled from the categorical rank list. Typically, this is used for fellowship programs offering clinical and research tracks, but reversion is an option for any fellowship. Reversion would be considered a limited solution; the process is unidirectional and requires one program to be prioritized over the other (e.g., combined over categorical) and does not allow for positions to revert across the distinct adult and pediatric subspecialty matches. The NRMP Institutional Official (IO) for the program must approve all reversions and they must be finalized and approved by the Rank Order List Certification Deadline for the match. More information on reversions can be found at http://www.nrmp.org/creating-reversions-fellowship-programs/. 

As an example, an adult ID program with three positions could opt to create one combined position and two categorical positions; the first position would preferentially be filled by a ranked combined candidate, but if unfilled would revert to a categorical position, allowing all three positions to be filled. 

## Discussion

Given the small numbers of residents eligible for and choosing to pursue combined adult and pediatric subspecialty training in a specific field, many residents and fellowship directors with a possible interest in pursuing combined fellowships lack needed information. We sought input from leadership representatives of stakeholders to summarize information relevant to the process, incorporate the perspective of trainees and fellowship directors, and highlight opportunities and barriers. 

There is limited existing literature outlining the experiences of other fellowships in developing combined programs. A description of the development of three combined clinical informatics and subspecialty fellowships is helpful in highlighting logistics of the ABMS petition process, addressing funding, and summarizing useful elements of the combined fellowship structure, including scholarly activity, electives, clinical time, and didactics [5]. A survey of specialists dually trained in adult infectious disease (ID) and critical care medicine (CCM) reported these combined specialists found their combination training and practice to be synergistic and satisfying and would reselect this training path, with 76% claiming they would retrain in ID and CCM [7]. 

The resident/applicant/fellow perspective

The National MedPeds Residents Association Fellowship guide offers a description of the many available combined subspecialty fellowships, as well as advice for how to approach the application process. Residents who have undertaken combined fellowships made recommendations to fellowship directors (Table 1).

**Table 1:** Perspectives and pearls from residents, applicants, and fellows

In considering a combined fellowship: When receiving inquiries regarding combined In In considering a combined fellowship: When receiving inquiries regarding combined internal medicine/pediatric fellowships, be direct and honest in interest and feasibility. If there is some interest in pursuing a combined fellowship position, let the potential applicant know and begin to explore scheduling possibilities and potential challenges with the counterpart program.
Maintain consistent and frequent contact with the applicant throughout the process. Much of this will be the responsibility of the applicant to stay in contact, but timely responses to questions and working through challenges that arise will improve the chances of a successful match.
In navigating the Match: Be consistent on which program the applicant should apply through (either internal medicine or pediatrics). Per NRMP policies, it is very difficult to switch between the two, and an applicant cannot apply to both simultaneously, so consistency and clarity regarding which Match path to apply through are appreciated.
Provide clarity and assurance in writing whenever possible. While not a legally binding agreement, having a plan set forth in writing, signed off by both departments, can do much to assuage fears and concerns, and prepares content for the ABMS petition.
During fellowship training: Semiannual meetings between the fellow and fellowship directors should include reviews of the combined curriculum and block schedule outlined in the ABMS petition, and be shared and confirmed with the combined-fellowship director.

The fellowship director perspective

The perspectives of fellowship directors are presented in Table 2.

**Table 2:** Perspectives and pearls from fellowship directors

	What are the key elements in planning a combined fellowship?
	“The critical pieces are a motivated FD and a motivated resident.” FDs suggested residents should be reaching out in the 3^rd^ of their 4-year training cycle to ask “are you willing” to allow time for an FD to develop a program option. FDs can demonstrate a willingness to work together across adult and pediatrics even if they do not currently offer a combined fellowship; for example, one FD noted before they formally offered a fellowship, their adult and pediatric divisions held co-conference once a week between our adult and peds [specialty] divisions and have experience producing research across those divisions.
	How do you approach the Match?
	We have a pre-match discussion and we’ve accommodated in a variety of ways, but most importantly it is an open conversation, and we provide a written letter of acceptance that is signed by all parties.
	How do you establish schedules?
	Some are clearly dictated by board requirements. The ABP yields 1 of the 3 years of pediatric ID training, but the remaining two we have no call and no clinic devoted to the “other side”. The overall schedule is sent to ABIM and ABP for approval within the first 6 months of the fellowship start (and the request can be made to the boards anytime after the Match) and it creates a clear outline of when they will be where. The reality is it has to be worked out on a case-by-case basis, but the fellows have been a great resource and worth the effort.
	How do you adjust the curriculum?
	We’ve made some curriculum changes on the research side because the pediatric boards are more stringent with the research requirement, but it isn’t a problem for the research to be pedi-focused and this allows it to be a continuum over 4 years and meet board guidelines. We have a month of Pedi ID each adult year and then similarly have a month of adult ID each pedi year, and that cross-pollination and mix in training is important for the combined fellows. We distinctly separate calls and clinic within those years – no pedi call or clinic on adult and vice versa.
	What challenges have you faced?
	The biggest complaint from fellows is of being pulled in two directions: is FD#1 frustrated when you’re on rotation with FD#2. But a lot of that is solved if you are explicit when you lay out the years and their content. Mostly the years we plan are distinctly adult or pediatric, with some co-conferences and clinics. It may weigh more heavily in smaller programs.
	What benefits do you see?
	We’ve had a number of fellows in combined training. There is not a lot we do differently; it is essentially the same for the adult fellows with 2 years of training, on the Pedi side it means changing from 3 to 2 years. They’re wonderful to recruit; the track record for combined fellows [is that they] are highly qualified and superb fellows.

## Conclusions

We hope and anticipate fellowship directors will meet applicants with unique qualifications and strong credentials that lead them to want to consider offering combined fellowship opportunities. If you are considering this option, you can reach out to your specialty fellowship organization to find peers who have experience and could provide guidance. The Medicine-Pediatrics Program Directors Association (https://www.im.org/about/governance/constituent-councils/mppda) can connect you with sites that have previously offered combined fellowship training in your specialty, and the National MedPeds Residents Association and its NMPRA Combined Fellowship Guide (https://medpeds.org/residents/fellowship-guide/) is an additional source of guidance for residents. 
